# Can clinical features be used to differentiate type 1 from type 2 diabetes? A systematic review of the literature

**DOI:** 10.1136/bmjopen-2015-009088

**Published:** 2015-11-02

**Authors:** Beverley M Shields, Jaime L Peters, Chris Cooper, Jenny Lowe, Bridget A Knight, Roy J Powell, Angus Jones, Christopher J Hyde, Andrew T Hattersley

**Affiliations:** 1 Department of NIHR Exeter Clinical Research Facility, University of Exeter Medical School, University of Exeter, Exeter, UK; 2 Department of Evidence Synthesis & Modelling for Health Improvement (ESMI), University of Exeter Medical School, University of Exeter, Exeter, UK; 3 Department of Research and Development, Royal Devon and Exeter NHS Foundation Trust, Exeter, UK

**Keywords:** STATISTICS & RESEARCH METHODS

## Abstract

**Objective:**

Clinicians predominantly use clinical features to differentiate type 1 from type 2 diabetes yet there are no evidence-based clinical criteria to aid classification of patients. Misclassification of diabetes is widespread (7–15% of cases), resulting in patients receiving inappropriate treatment. We sought to identify which clinical criteria could be used to discriminate type 1 and type 2 diabetes.

**Design:**

Systematic review of all diagnostic accuracy studies published since 1979 using clinical criteria to predict insulin deficiency (measured by C-peptide).

**Data sources:**

14 databases including: MEDLINE, MEDLINE in Process and EMBASE. The search strategy took the form of: (terms for diabetes) AND (terms for C-Peptide).

**Eligibility criteria:**

Diagnostic accuracy studies of any routinely available clinical predictors against a reference standard of insulin deficiency defined by cut-offs of C-peptide concentrations. No restrictions on race, age, language or country of origin.

**Results:**

10 917 abstracts were screened, and 231 full texts reviewed. 11 studies met inclusion criteria, but varied by age, race, year and proportion of participants who were C-peptide negative. Age at diagnosis was the most discriminatory feature in 7/9 studies where it was assessed, with optimal cut-offs (>70% mean sensitivity and specificity) across studies being <30 years or <40 years. Use of/time to insulin treatment and body mass index (BMI) were also discriminatory. When combining features, BMI added little over age at diagnosis and/or time to insulin (<1% improvement in classification).

**Conclusions:**

Despite finding only 11 studies, and considerable heterogeneity between studies, age at diagnosis and time to insulin were consistently the most discriminatory criteria. BMI, despite being widely used in clinical practice, adds little to these two criteria. The criteria identified are similar to the Royal College of General Practitioners National Health Service (RCGP/NHS) Diabetes classification guidelines, which use age at diagnosis <35 years and time to insulin <6 m. Until further studies are carried out, these guidelines represent a suitable classification scheme.

**Systematic review registration:**

PROSPERO reference CRD42012001736.

**Strengths and limitations of this study**

- We have carried out a comprehensive and robust systematic review in accordance with PRISMA guidelines and our initial published protocol.
- We screened a large number of literature sources, and all reviewing and data extraction was carried out in duplicate independently by two authors (BS and JP).
- Considerable heterogeneity across studies precluded a formal meta-analysis.
- A limited number of studies were found meaning there is still considerable uncertainty around criteria for classification of type 1 and type 2 diabetes.
- Variability in the reference standard of insulin deficiency across studies also led to further uncertainty around findings limiting direct usefulness of criteria.

## Background

Correct classification of a patient's diabetes is crucial for ensuring they receive the most appropriate treatment and management. Current guidelines for the treatment of diabetes are specific to type 1 and type 2 diabetes (T1D and T2D) and these show marked differences,^1–4^ reflecting the difference in endogenous insulin production between the two subtypes. Patients with T1D rapidly develop severe insulin deficiency, leading to high glycemic instability and so require accurate insulin replacement (such as multiple injections and carbohydrate counting), and have poor response to non-insulin therapies.^3^ ^5^ Patients with T2D still continue to produce substantial amounts of their own insulin, and, therefore, respond to non-insulin therapy, have more stable glycaemia and, if insulin treatment is needed, may achieve good control with non-physiological insulin regimes.^6^ ^7^

Currently, there are no published, evidence-based, guidelines or criteria for diabetes classification, despite the importance for patient management. Guidance on the classification of the two types of diabetes from major health organisations is limited, and focuses on aetiology,^8^ ^9^ whereas it is insulin production that is the driver for informing treatment decisions. Insulin deficiency/production can be assessed by measurement of C-peptide in either blood or urine,^10^ but it is rarely measured in clinical practice and current guidelines for diabetes management do not recommend its routine use.^1^ ^3^ ^11^ Classification is based primarily on clinical judgement, with younger slimmer patients tending to be classed as T1, and older, more obese patients diagnosed as T 2.^8^ However, with obesity increasing in the population and the resulting increase in T2D in the young, this traditional distinction has become less clear.^12^ ^13^

Misclassification of diabetes has been shown to occur in 7–15% of cases,^13–15^ and these studies are likely to underestimate the problem, as they only use clinical ‘clues’ as their reference standard. The current practice based on aetiological guidelines and clinical opinion is clearly insufficient. Pragmatic guidelines on diabetes classification have been developed by National Health Service (NHS) Diabetes and The Royal College of General Practitioners (RCGP) in the UK, but are taken from consensus expert clinical opinion rather than being evidence-based.^13^

In order to determine evidence-based criteria that could be used to classify the two main forms of diabetes, an appropriate gold standard is necessary. The most important reason for correctly classifying patients is to ensure appropriate treatment and management, and the main factor determining this is the difference in endogenous insulin production between patients with T1 and T2D. Therefore, long-term insulin deficiency represents an acceptable reference standard for T1D. This is likely to be preferable to using markers of the autoimmune process associated with T1D. While measurement of various islet autoantibodies may aid discrimination, these are imperfect measures,^16^ and most importantly, the presence of islet autoimmunity does not in itself determine treatment requirement.^17^

We aimed to systematically review the literature to identify clinical criteria, predictive of severe insulin deficiency, that could be used to discriminate T1D and T2D and inform evidence-based guidelines for the classification of diabetes.

## Methods

We followed the PRISMA guidelines for the reporting of systematic reviews. The original protocol has been published^18^ and is registered on PROSPERO (http://www.crd.york.ac.uk/PROSPERO/reference CRD4201200173 6).

### Data sources and search strategy

Fourteen databases were searched systematically: MEDLINE, MEDLINE in Process, EMBASE, PsycINFO, Social Policy and Practice, AMED, British Nursing Index, CINAHL, HMIC, Sociological Abstracts, ASSIA, Cochrane, Web of Science, Centre for Reviews and Dissemination). The search strategy took the form of: (terms for diabetes) AND (terms for C-Peptide). Searches were limited to human only populations and from 1979 since that was when the original classification scheme was proposed by the National Diabetes Data Group.^19^ Searches were not limited by language or study design.

Searches were also carried out on the Conference Proceedings Citation Index as well as the proceedings of the American Diabetes Association, the European Association for the Study of Diabetes, and Diabetes UK. BL Ethos was also searched for theses. Web-searching was conducted, including web-site specific searches of WHO and NICE. Forwards and backwards citation chasing was conducted on all studies included at full-text. The full search strategies are recorded in the online supplementary Search Annex. Searches were initially performed in October 2012 and were updated on 3 April 2014 to capture any additional studies that may have been carried out since the beginning of the review.

### Study selection

A two-stage screening process was undertaken. In Stage 1, after removing duplicates, two reviewers (BMS and JLP) independently screened the titles and abstracts of all references against the inclusion and exclusion criteria. In Stage 2, full texts were retrieved on all studies included at the first screening stage and were independently screened (by BMS and JLP). Authors of included conference abstracts were searched to determine whether a full article had subsequently been published. Any discrepancies between the two reviewers were discussed and resolved by consensus, or in discussion with a third reviewer (RJP).

### Inclusion and exclusion criteria

Included studies comprised diagnostic accuracy studies of clinical predictors of insulin deficiency, with the reference standard of insulin deficiency being defined by cut-offs of C-peptide results. All measurements of C-peptide and all cut-offs for insulin deficiency were included. Clinical predictors were defined as any routinely measured clinical feature and studies were eligible if there was a cut-off for that clinical predictor assessed against the measure of insulin deficiency. There were no restrictions on race, age or country of origin. Studies examining islet autoantibodies only were excluded as they are not routinely measured. A separate systematic review examining the diagnostic accuracy of islet autoantibodies is presently underway (Prospero reference CRD42012001736). Studies where patients had known causes of diabetes, for example, monogenic, secondary or syndromic diabetes, were excluded.

### Data extraction

For all studies meeting the inclusion and exclusion criteria, data were extracted independently by both reviewers (BMS and JLP). Data extraction forms were developed and piloted prior to the review. Key details of population (age, sex, country, race, year), diabetes (definition of diabetes, treatment, subgroups), reference standard (type of sample, stimulation, assay, cut-off used) and clinical predictors (which predictors were included, how they were measured, the cut-offs used) were recorded. All C-peptide cut-offs were converted to the fasting serum equivalent to allow direct comparison.^10^ Two-by-two tables were extracted where possible to determine the proportion of patients who were C-peptide negative/positive (ie, below/above the cut-off) and the sensitivity, specificity, positive and negative predictive values of the clinical characteristics at reported cut-offs.

### Quality assessment

Both reviewers (BMS and JLP) assessed quality independently and discrepancies were resolved by consensus. Quality assessment forms, based on the criteria set out in QUADAS-2,^20^ were developed and piloted prior to review. These criteria included assessment of internal and external validity of patient selection, the clinical predictors and patient flow and timing. Variability in the measurements for the reference standard was assessed separately. Further details are available in the online supplementary material.

### Data synthesis

Owing to the considerable heterogeneity between the studies identified, meta-analysis, as proposed in our original protocol, was not appropriate. Data synthesis is, therefore, largely descriptive with summary data presented. Criteria with a mean of sensitivity and specificity >70% (equivalent to a receiver operating characteristic area under curve of 0.7) were considered clinically useful. Ranking of the discriminatory ability of criteria *within* studies was used to compare their relative performance.

### Reporting bias

No formal assessment of publication bias was undertaken due to heterogeneity between studies and the small number of included studies. We did perform a comprehensive and exhaustive search including grey literature, however it cannot be ruled out that our systematic review is affected by reporting biases.

## Results

### Initial screening

**Figure 1:**
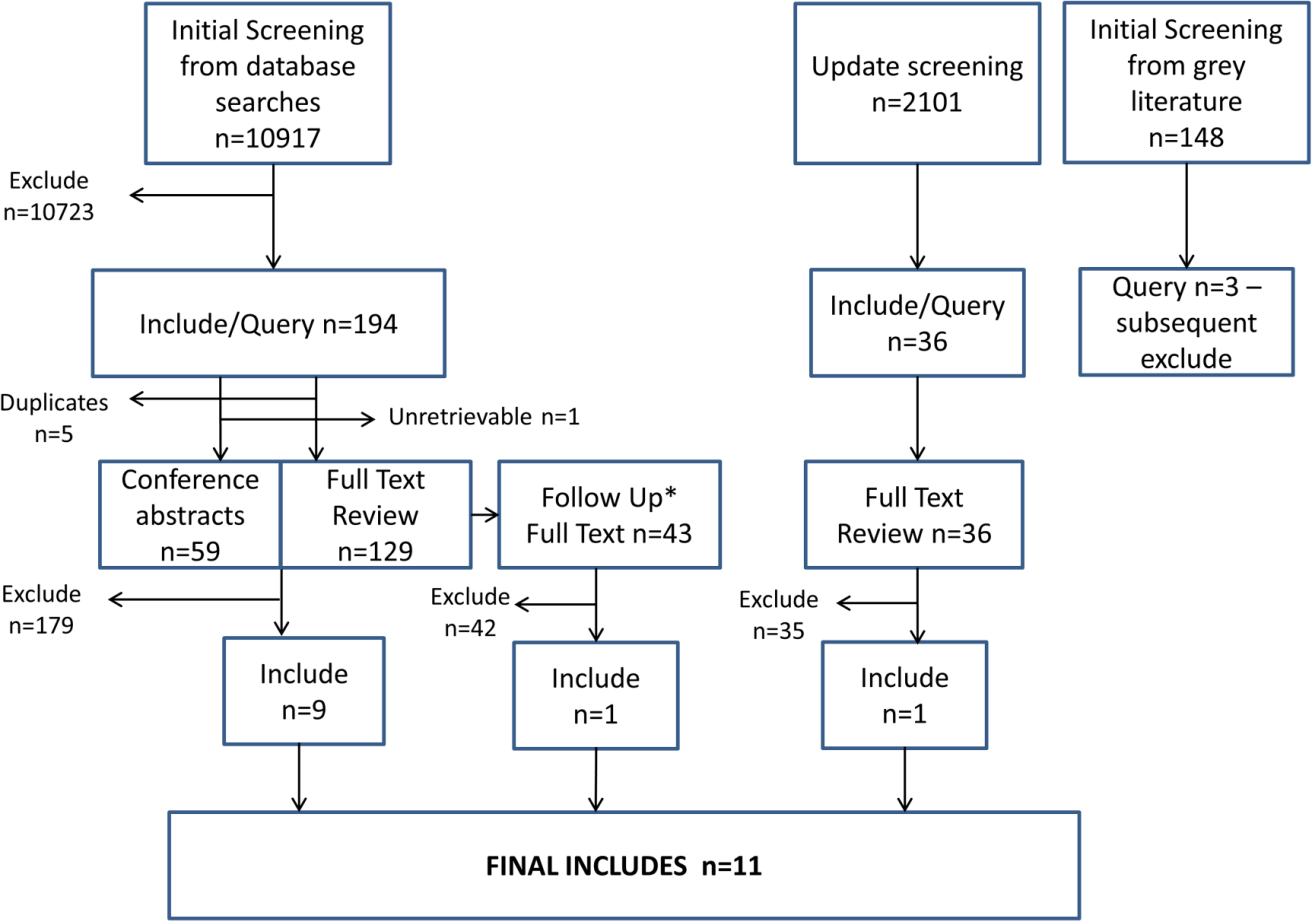
Flow diagram showing inclusions and exclusions from title and abstract screening, and full-text review. *Follow-Up includes full texts identified from follow-up of conference abstracts (n=29) and references identified from backwards and forwards citation chasing (n=14).

Figure 1 shows the flow diagram of citations found. A total of 10 917 records were identified from database searches and a further 148 sources were identified from grey literature searches. After title and abstract screening, 194 articles were deemed potentially relevant. Following full-text screening, nine studies were identified as eligible based on our inclusion criteria^21–29^ (for further details see online supplementary material).

Backward and forward citation searching was carried out on the nine included references, and conference abstracts were followed up, identifying a further 43 studies for full-text review, one of which^30^ met our inclusion criteria. In April 2014, an update search was performed yielding a further 2101 references for screening. Thirty-six of these were identified by the two reviewers as requiring full-text review, and one of these fitted inclusion criteria.^31^ Thus, 11 articles contribute to this systematic review.

### Data extraction and quality assessment

There was considerable heterogeneity across the included studies (see online supplementary table S1). The 11 included studies spanned a wide range of years (1981–2013). Studies varied in terms of race, age group and subgroups of diabetes studied. One study included only patients with end-stage renal disease,^22^ whereas it was a specific exclusion criterion for another study.^28^ Three studies focused on insulin-treated patients only,^24^ ^29^ ^30^ whereas the other studies either included all patients regardless of treatment or did not report on treatment. Sample size ranged from <100^22^ ^29^ ^31^ to >3000.^25^ The proportion of patients classified as insulin deficient (based on the reported C-peptide cut-off in each paper) also varied (median (range) 40% (7–69%)), reflecting differing inclusion criteria across studies altering the proportions with different forms of diabetes across the studies.

Quality assessment of the included studies is summarised in online supplementary table S2. In general, there was a low risk of bias in terms of patient selection and patient flow/timing. Two studies were at high risk of bias^22^ ^29^ in terms of the clinical criteria used as these were internally derived, so diagnostic performance is likely overestimated in these cases.^32^ In terms of external validity, studies were all applicable to our broad research question but most restricted inclusion criteria to a subset of the diabetic population.

The reference standards varied in terms of sample provided, timing of sample in relation to meal stimulation, and cut-offs for C-peptide (see online supplementary table S3). Five studies report deriving their cut-offs from previous papers.^21^ ^22^ ^25^ ^29^ ^30^ Two studies derived the cut-off used from their own data,^27^ ^28^ potentially introducing bias, although the cut-offs were comparable to those derived from the literature. Despite the variation in measurements, all were appropriate to classify insulin deficiency and cut-offs were largely comparable with most approximating 0.2 nmol/L,^21^ ^22^ ^24–26^ ^28^ ^30^ and four studies using a slightly more conservative cut-off (0.03–0.08 nmol/L). 23 27 29 31 Only one study measured C-peptide and clinical features at diabetes diagnosis.^31^ All other studies were cross-sectional with varying duration of diabetes.

### Data synthesis

Owing to the heterogeneity across studies, particularly in terms of inclusion criteria, formal quantitative meta-analysis was not appropriate. Therefore, data synthesis is largely descriptive.

### Age at diagnosis, BMI, insulin treatment/time to insulin are consistent predictors of insulin deficiency across studies

Age at diagnosis (9 studies), measures of obesity (including BMI, or percentage desirable weight in earlier studies) (8 studies) and either time to insulin treatment (5 studies) and/or use of insulin treatment (3 studies) were identified as consistent clinical criteria predictive of insulin deficiency (table 1). In all studies reporting these criteria, younger age at diagnosis, slimmer BMI and shorter time to insulin was used to define insulin deficiency.

**Table 1:** Criteria reported in the 11 included studies used to discriminate between C-peptide positive and negative patients

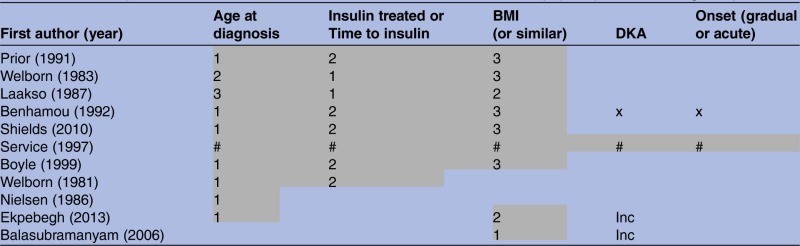

Numbers indicate their ranking in terms of discriminatory ability within studies, with 1 representing the most discriminatory. # indicates used as part of an algorithm, but discriminatory value of individual criteria not reported. × indicates features not discriminatory. ‘Inc’ indicates inclusion criteria for the study, so feature could not be used to discriminate. Only features reported in more than one paper shown (see text for details of others).

BMI, body mass index; DKA, diabetic ketoacidosis.

Absence of each of acanthosis nigricans and hypertension were predictive of insulin deficiency (overall correct classification rates of 61% and 72%, respectively), but these were only assessed in one study.^31^ Other measures were available in four studies^22^ ^26^ ^27^ ^31^ (including history of diabetic ketoacidosis (DKA)^22^ ^26^ or ketonuria,^22^ history of hypoglycaemia,^27^ speed of onset of diabetes,^26^ long-term complications,^22^ polyuria,^22^ weight loss,^22^ post-Sustacal glucose,^27^ serum creatinine,^27^ diabetes in a first-degree relative^31^ and history of poor control^27^), but they were either not discriminatory, or they contributed very little individual discriminatory power to an overall algorithm.

### Age at diagnosis cut-offs better predicted insulin deficiency than cut-offs of BMI or time to insulin

When comparing discriminative ability of the most commonly reported criteria *within* studies, age at diagnosis, at the cut-off described in the individual study, correctly classified more patients than the other clinical features (most discriminatory criteria in 7/9 studies). Time to insulin/insulin treatment was the next best predictor, and BMI (or equivalent) was the weakest of the significant predictors (table 1).

### Cut-offs for age at diagnosis, BMI and time to insulin were fairly consistent across studies

Cut-offs with the best combination of sensitivity and specificity (mean of sensitivity and specificity >70%) were similar across studies. For predicting insulin deficiency, the best cut-offs for age at diagnosis were <30 years (2 studies) or </≤40 years (4 studies). For time to insulin, <1 year (1 study) or </≤2 years (2 studies) were the best cut-offs, although longer cut-offs were not assessed in any of the studies identified. For BMI, cut-offs <27 kg/m^2^ (1 study) and <28 kg/m^2^ (3 studies) were most useful (see table 2). Extracted 2×2 tables are presented online supplementary tables 4.

**Table 2:** Criteria for predicting type 1 diabetes—single criteria

Cut-off	Author (year)	N	Per cent C-pep neg	Sens (%)	Spec (%)	Mean sens and spec	Per cent correct	PPV	NPV
*(i) Age at diagnosis (a/d)*									
<20	Boyle (1999)	3613	7	20	97	59	92	36	94
**≤30**	**Prior (1991)**	**575**	**61**	**84**	**82**	**83**	**83**	**88**	**77**
**<30**	**Nielsen (1986)**	**215**	**69**	**64**	**88**	**76**	**72**	**92**	**53**
<30	Ekpebegh (2013)	71	49	57	72	65	65	67	63
**<39**	**Shields (2010)**	**72**	**56**	**68**	**97**	**83**	**81**	**96**	**70**
**≤40**	**Prior (1991)**	**575**	**61**	**97**	**59**	**78**	**82**	**79**	**92**
**≤40**	**Welborn (1983)**	**121**	**21**	**84**	**85**	**85**	**85**	**60**	**95**
**≤40**	**Welborn (1981)**	**201**	**24**	**76**	**81**	**79**	**79**	**55**	**92**
≤40	Laakso* (1987)	171	67	61	79	70	67	85	44
<45	Boyle (1999)	3613	7	65	57	61	57	10	96
*(ii) Insulin treatment/time to insulin (tti) (a=all treatments, i=insulin-treated only)*									
on insulin (a)	Prior (1991)	575	61	99	25	62	70	68	97
**on insulin (a)**	**Welborn (1981)**	**201**	**24**	**100**	**70**	**85**	**77**	**49**	**100**
**on insulin (a)**	**Boyle (1999)**	**3613**	**7**	**91**	**61**	**76**	**63**	**15**	**99**
tti≤1.5 m (i)	Shields (2010)	72	56	80	56	68	69	70	69
**tti<1y (a)**	**Prior (1991)**	**575**	**61**	**92**	**75**	**84**	**85**	**85**	**85**
**tti<2y (a)**	**Welborn (1983)**	**121**	**21**	**100**	**82**	**91**	**86**	**60**	**100**
**tti≤2y (i)**	Laakso* **(1987)**	**90**	**67**	**70**	**86**	**78**	**75**	**91**	**58**
*(iii) BMI*									
<20	Boyle (1999)	3613	7	10	98	54	92	33	94
<25†	Prior (1991)	575	61	34	92	63	57	87	47
<25	Boyle (1999)	3613	7	41	86	64	83	18	95
**<27**†	**Prior (1991)**	**575**	**61**	**87**	**63**	**75**	**78**	**79**	**76**
**≤27**†	**Welborn (1983)**	**121**	**21**	**80**	**67**	**74**	**69**	**38**	**93**
**≤27**	Laakso* (1987)	**90**	**67**	**76**	**66**	**71**	**73**	**82**	**57**
**<28**	**Balasumbryaman (2006)**	**294**	**60**	**67**	**86**	**77**	**78**	**79**	**77**
<29	Boyle (1999)	3613	7	71	57	64	58	11	96
<29	Shields (2010)	72	56	78	56	67	68	69	67
<30	Ekpebegh (2013)	71	49	77	47	62	62	59	68

Sensitivity (sens), specificity (spec), proportion correctly classified (%correct), mean of sensitivity and specificity (mean sens and spec), positive predictive value (PPV), and negative predictive value (NPV) for (i) age at diagnosis, (ii) body mass index (BMI) and (iii) insulin treatment and/or time to insulin. Proportion of C-peptide negative patients (% C-pep neg) shown to aid interpretation of % correct, PPV and NPV. Criteria with a mean sensitivity and specificity >70% are highlighted in bold.

*Male and female values combined, using postglucagon-stimulated results.

†Converted from percentage desirable weight.

### BMI cut-offs provide little improvement in classification in addition to age at diagnosis and insulin use/time to insulin criteria

Combinations of cut-offs did not consistently improve the overall rate of classification. The addition of BMI did not improve classification over age at diagnosis and/or use of/time to insulin treatment in all five studies where these combinations were reported (<1% improvement in classification; see table 3). The addition of insulin treatment or time to insulin criteria improved classification over using age at diagnosis alone in 3/5 studies where both were reported (see table 3). Extracted 2×2 tables and summary statistics are presented in online supplementary tables S4 and S5.

**Table 3:** Comparison of combinations of criteria over individual criteria.

Author (year)	N	Individual Criteria Per cent correctly classified			Combined—2 criteria Per cent correctly classified			Combined—3 criteria Per cent correctly classified	
		Age at diagnosis	BMI (or equivalent)	Insulin treatment/ Time to insulin (TTI)	Age at diagnosis and BMI	Age at diagnosis and Insulin/TTI	BMI and Insulin/TTI	Age at diagnosis, BMI and Insulin/TTI	Regression equation or algorithm using all 3 criteria
Boyle (1999)	3613 (1807†)	92	58	63		90		93	93
Laakso (1987)	171	67	73	75	61	61	67	56	
Prior (1993)	575	82	78	85		89***		80	89
Shields (2012)	72	81	68	69				82	
Welborn (1981)	203	79		77		88**			
Welborn (1983)	121	85	69	86		93**			93

Data presented as overall percentage correctly classified according to C-peptide category (below or above cut-off for insulin deficiency) using cut-offs of individual criteria and combinations of criteria, for the six studies where comparison within studies was possible. Results in bold are those where the addition of another clinical feature provides better classification within studies.

**p<0.01, ***p<0.001, by McNemar's test.

†Regression equations/algorithms tested on a separate data set, so a two sample χ^2^ test is used to determine statistical significance.

BMI, body mass index.

## Discussion

### Principal findings

#### Few studies have robustly assessed utility of clinical features in diagnosing diabetes subtype

There were only 11 appropriate studies that examined which clinical characteristics could discriminate between T1 and T2D, using the reference standard of insulin deficiency. This is a remarkably low number of studies considering the vast majority of the >200 million patients with diabetes will be classified into type 1 or type 2 on the basis of clinical features alone and an incorrect classification will result in inappropriate treatment.

#### Age at diagnosis was the most discriminatory clinical feature

Age at diagnosis, time to insulin and BMI consistently emerged as the main discriminatory clinical criteria despite the considerable heterogeneity of the included studies. Age at diagnosis was the best discriminatory criteria with diagnosis either below 30 or below 40 years being predictive of T1D. In terms of providing useful criteria for clinical practice, based on the current available evidence, this would suggest clinicians should place more emphasis on age than obesity when diagnosing diabetes subtype, but exercise caution when classifying patients diagnosed between the ages of 30 and 40 where further investigation is likely to be necessary.

#### Time to insulin treatment is a useful discriminator, but biased by physician opinion

Starting insulin treatment before 2 years did slightly improve discrimination over age of diagnosis (table 3). However, treatment assignment can clearly not be used to define initial treatment, which is one of the major reasons for determining diabetes subtype. Treatment decisions are physician-dependent, as well as disease-dependent, so will vary between clinicians.

#### BMI discriminatory but adds little over age at diagnosis

BMI provided <1% improvement in classification over age at diagnosis or age at diagnosis and time to insulin. Clinicians often use obesity as a marker to indicate T2D, but our findings suggest using this is unlikely to be helpful over and above using age at diagnosis.

#### Other may not be sufficiently discriminatory

Other measures were less often studied. Acanthosis nigricans and hypertension did discriminate C-peptide positive from C-peptide negative patients, but these were only assessed in one study. Other features were either not discriminatory or only contributed weakly to an algorithm, and therefore unlikely to be useful in practice. These measures included features of diagnosis such as diabetic ketoacidosis, ketonuria and rapid onset of symptoms including weight loss. In fact, in the two studies examining only patients presenting with DKA, 40% and 46% were C-peptide positive,^21^ ^31^ suggesting DKA is not useful in its own right for classifying a patient as having type 1 diabetes.

### Strengths and weaknesses

#### Strengths

We have carried out a comprehensive and robust systematic review in accordance with PRISMA guidelines and our initial published protocol.^18^ We screened a large number of literature sources, and all reviewing and data extraction was carried out in duplicate independently by two authors (BMS and JLP).

#### Limitations

Heterogeneity across studies could have influenced the diagnostic performance of cut-offs identified and so precluded formal meta-analysis. There were four key areas in particular, where heterogeneity was apparent: (1) The proportion of insulin-deficient patients varied considerably across the studies (range 7–69%), reflecting major differences in inclusion criteria for each study and varying proportions of T1 and T2D in the study populations. (2) Studies spanned over 30 years (1981–2013) and there have been considerable changes in the phenotype of T1 and T2D in this time. With the rising prevalence of obesity in the population, T1 patients are now more likely to be obese than in the past, and T2D has become more common in young adults. (3) Renal disease is known to impact on C-peptide clearance, so differences were likely in the studies excluding patients with renal disease,^25^ ^28^ compared with those exclusively examining those with ESRD.^22^ (4) Ethnicity differed across studies, from populations that were predominantly Caucasian,^23^ ^27^ to those predominantly Hispanic and/or Black African^31^/African-American patients.^21^ ^25^ Despite the considerable differences in studies, however, there were consistencies in the criteria identified and the most discriminatory cut-offs across the different populations.

The small number of studies and the heterogeneity between them means there is still uncertainty around the usefulness of the criteria and cut-offs proposed, and highlights a clear need for further work in this area. This review provides a strong starting point from which to develop future prediction criteria.

Differences in the reference standards (eg, in the samples, stimuli, assay used and cut-offs used) highlighted problems with our reference standard for T1D. However, although cut-offs were derived in a variety of ways, they were largely comparable and appropriate for detecting insulin deficiency in the populations of interest. Where more than one cut-off was used,^24^ ^26^ ^27^ ^30^ this made little difference (<12%) to the proportion of patients classified and the cut-offs identified. These differences represent potential issues with using our ‘gold standard’ for insulin deficiency when aiming to classify T2D. We would therefore suggest caution in future studies when classifying patients close to the proposed C-peptide cut-off.

#### Other forms of diabetes

We have only considered the two main forms of diabetes for which there are clear national and international treatment guidelines. Rarer subtypes are not considered here. Other forms of diabetes, such as latent autoimmune diabetes of adults, are not included in international guidelines and appropriate treatment would be guided by insulin deficiency, our gold standard. Further work would be needed to derive criteria for a ‘grey area’ where diagnosis of subtype is less certain and further investigations would be required to aid classification.

### Implications and future work

Evidence-based guidelines on the classification of T1D and T2D need to include clinical criteria on how the diagnosis should be made. This is a major omission in current national and international guidelines for diabetes. The evidence as identified in this review suggests age at diagnosis and time to insulin (when available) are essential components as they contribute most to the predictive ability. BMI, and other clinical criteria, do not appear to add to add further discrimination. The criteria identified are similar to the RCGP/NHS Diabetes Guidelines for Classification^13^ which are based on consensus expert opinion. These guidelines would therefore, represent a suitable classification scheme until a stronger evidence base is available.

New studies are urgently needed to further develop and validate criteria suitable for classifying diabetes. We identified no studies in the Asian or paediatric populations, and only one study assessing features close to diagnosis.^31^ Determining classification rules for both the incident and prevalent population would be important. Labelling a patient's diabetes at the outset is crucial as the classification given is rarely reconsidered. The evidence in this review should be used to redevelop a clinical prediction tool for T1D and T2D. C-peptide is likely to be less discriminatory at diagnosis, as patients with T1D can still produce their own insulin in the ‘honeymoon’ period, so it would be important to examine predictors of insulin deficiency after this time. Future studies should be large-scale, prospective and give results for all racial and age groups using follow-up C-peptide measurements at least 3 years after diagnosis as an outcome. These studies would help answer if clinical criteria used in combination are sufficient to accurately classify diabetes, or whether investigations, such as islet autoantibodies, are needed in addition. Consideration of other forms of diabetes, such as monogenic diabetes, is also important.

We did not include antibodies in our search criteria as we limited our review to routinely available clinical criteria. Antibodies may represent a useful test at diagnosis, where C-peptide is of limited value due to the ‘honeymoon period’, where patients with T1D are still able to produce significant amounts of their own insulin for a short period of time. A systematic review examining the use of antibodies at predicting long-term insulin deficiency is presently in progress (Prospero reference CRD42012001736)

In conclusion, we have performed the first systematic review of the literature that examines using clinical criteria for the classification of diabetes. Although, only 11 studies were identified, age at diagnosis and time to insulin were consistent as discriminatory criteria across studies. BMI did not aid classification over these factors. The discriminatory criteria identified were similar to those proposed by the RCGP/NHS Diabetes Classification guidelines, so these would represent a suitable classification scheme at present. New studies are urgently needed to assess and validate the most appropriate clinical criteria. This review provides a summary of the current knowledge base for reference in any future studies developing classification rules.
